# Opioid receptor agonist–antagonists in pain management: receptor specific mechanisms, dose response relationships, and clinical combination strategies

**DOI:** 10.3389/fpain.2026.1903184

**Published:** 2026-08-21

**Authors:** Sining Zhao, Zhiqiang Xue, Shu Wang

**Affiliations:** 1 Department of Anesthesiology, Benxi Central Hospital, Benxi, Liaoning, China; 2 The Third Clinical Medical College, Jinzhou Medical University, Jinzhou, Liaoning, China

**Keywords:** analgesic ceiling effect, dose-response relationship, intrinsic efficacy, multimodal analgesia, opioid receptor agonist-antagonists, receptor selectivity

## Abstract

Opioid receptor agonist–antagonists occupy a distinctive position between high-efficacy agonists and pure antagonists and have been widely used in clinical pain management. Nevertheless, their pharmacological complexity and dose-dependent receptor interactions have led to persistent controversy regarding their analgesic efficacy, ceiling effects, and clinical performance when combined with full *μ*-opioid receptor agonists. Most existing reviews focus on individual agents or isolated mechanisms and do not adequately address the dynamic relationship between receptor selectivity, intrinsic efficacy, and dose–effect behavior that underlies their clinical outcomes. This narrative review integrates recent preclinical and clinical evidence to examine the pharmacological properties and clinical application strategies of opioid receptor agonist–antagonists, with particular emphasis on *κ*-opioid receptor agonism, μ-opioid receptor partial agonism or antagonism, and their interactions across different dose ranges. We discuss the mechanistic basis of the analgesic ceiling effect observed with μ-partial agonists, highlighting that this phenomenon reflects limited intrinsic efficacy rather than a dose-dependent transition to pure antagonism. Furthermore, the review analyzes how agonist–antagonists may exert synergistic or functional antagonistic effects when co-administered with full μ-opioid agonists, depending on dose, receptor affinity, and binding kinetics. At low to moderate doses, complementary *κ*–μ receptor modulation may enhance analgesia while attenuating opioid-related adverse effects such as respiratory depression, pruritus, and nausea, whereas excessive dosing may compromise analgesic efficacy or increase sedation. By synthesizing receptor-specific mechanisms with clinical dosing considerations, this review proposes a receptor-selective, dose-dependent framework to support safer and more effective use of opioid receptor agonist–antagonists in contemporary multimodal pain management.

## 1 Introduction

Opioids remain a cornerstone of pharmacological pain management across perioperative, cancer-related, and chronic pain settings. Despite their well-established analgesic efficacy, the widespread use of full μ-opioid receptor agonists is limited by dose-dependent adverse effects, including respiratory depression, nausea, constipation, tolerance, and the risk of misuse and dependence (1). These challenges have driven continued interest in opioid-sparing strategies and in alternative opioid formulations that aim to balance effective analgesia with improved safety.

Opioid receptor agonist–antagonists occupy a unique position within the opioid pharmacological spectrum. Unlike full agonists, these agents exhibit mixed intrinsic activities across opioid receptor subtypes (2). In clinical practice, agonist–antagonists have been used for decades in anesthesia and pain medicine, often with the expectation of a lower risk of respiratory depression and abuse. However, their clinical use remains inconsistent, and their analgesic performance, particularly at higher doses or when combined with full μ-opioid agonists, has been a persistent source of controversy.

One of the most controversial characteristics of opioid agonist-antagonists is the so-called analgesic ceiling effect, which is generally considered that analgesic efficacy no longer improves with increasing drug doses (3–5). This concept has often been oversimplified, leading to the assumption that these agents behave as pure antagonists beyond a certain dose threshold. In reality, the ceiling effect may reflect complex interactions between receptor selectivity, intrinsic efficacy, and dose–response dynamics, rather than a uniform pharmacological switch (4–6). Misinterpretation of this phenomenon has contributed to suboptimal dosing strategies and inconsistent clinical outcomes.

Further complexity arises when opioid agonist–antagonists are administered in combination with full μ-opioid receptor agonists, a scenario that is increasingly common in multimodal analgesia (7). Depending on dose, receptor affinity, and binding kinetics, agonist–antagonists may enhance analgesia through complementary receptor modulation or attenuate opioid effects through functional antagonism. Understanding these interactions is essential for rational combination therapy but remains inadequately addressed in many existing reviews, which often focus on individual agents rather than shared pharmacological principles.

In this narrative review, we synthesize current preclinical and clinical evidence to examine the pharmacological properties and clinical application strategies of opioid receptor agonist–antagonists. Emphasis is placed on receptor subtype selectivity, intrinsic efficacy, and dose-dependent effects, with particular attention to their implications for combination therapy with full μ-opioid agonists. By integrating mechanistic insights with clinical considerations, this review aims to provide a receptor-selective, dose-dependent framework to support safer and more effective use of opioid agonist–antagonists in contemporary pain management.

## 2 Opioid receptor agonist–antagonists

Research on opioid receptors originated from pharmacological observations of heterogeneous responses to opioid drugs. According to the review by Martin (1979), the development of opioid agonist–antagonists can be traced back to Pohl's investigation of nalorphine in 1915. Subsequent synthesis and characterization of nalorphine demonstrated its ability to antagonize morphine effects while precipitating withdrawal symptoms, thereby stimulating continued exploration of mixed-activity opioids. Later studies revealed that although these agents exhibited potent analgesic properties, they were also associated with adverse reactions such as dysphoria and agitation, which in turn promoted the development of newer compounds exemplified by pentazocine (8).

Opioid receptor agonist-antagonists can be defined as a class of drugs that exert mixed and selective actions on opioid receptor subtypes. They typically act as agonists at *κ*-opioid receptors while functioning as antagonists or partial agonists at μ-opioid receptors. This definition derives from a profound understanding of receptor pharmacology and clinical effects and has been widely accepted in the fields of medicine and pharmacy globally. The opioid receptor family comprises μ, *δ*, *κ*, and NOP (nociceptin/orphanin FQ receptor, formerly ORL1); *σ* receptors are not opioid receptors (9). The receptors primarily relevant to analgesia and adverse effects in clinical practice are μ, *κ*, and *δ* receptors. However, existing clinically available agonist-antagonist drugs have little to no activity at *δ* receptors (10). Pure *δ*-receptor agonists remain at the preclinical research stage; although they possess analgesic effects with virtually no respiratory depression and extremely low addictive potential, they are prone to inducing convulsions and have not yet been approved for clinical use. NOP receptor signaling has context-dependent effects on pain. NOP agonism may oppose opioid analgesia after supraspinal administration in some rodent models, but can also produce potent antinociception depending on the site of administration, species, dose, and pain assay (11). Accordingly, NOP should not be characterized as uniformly non-analgesic. Buprenorphine, a clinically used mixed opioid ligand, also has weak/low-affinity agonist activity at NOP/ORL1, although μ-opioid receptor activation remains the principal mediator of its clinical analgesia (11, 12). This pharmacological profile has stimulated development of bifunctional MOP/NOP ligands such as BU08028, which produced potent, long-lasting antinociception with low reinforcing effects and no respiratory depression at tested doses in nonhuman primates; these compounds remain investigational (13). Thus, NOP-directed mechanisms have therapeutic promise but are not yet established as routine clinical targets for this drug class.

Beyond the *σ* receptor-mediated effects noted above, activation of the *κ*-opioid receptor itself can produce dose-dependent dysphoria, anxiety, and hallucinations in susceptible individuals. These effects are mediated through *κ*-receptor signaling in limbic and cortical regions, where receptor activation modulates dopamine release and emotional processing, generating aversive states that functionally oppose the rewarding effects of μ-receptor stimulation. Importantly, while this property contributes to the reduced abuse liability of *κ*-agonists, it also imposes a significant clinical burden, particularly at higher doses, and necessitates careful patient selection and dose titration. The psychotomimetic effects are most prominent with agents possessing strong *κ*-agonistic activity, such as pentazocine, whereas agents with weaker *κ* efficacy or additional μ-partial agonism may exhibit a more favorable profile.

Therefore, in clinical contexts, opioid receptor agonist-antagonists are often narrowly defined as *κ*-opioid receptor agonists that simultaneously exert antagonistic or partial agonistic effects at μ-opioid receptors. When administered as monotherapy, these agents exert clinically meaningful μ-receptor antagonistic activity despite the absence of exogenous μ-opioid agonists to oppose. By directly limiting μ-receptor–mediated signaling, this mechanism is hypothesized to attenuate the neurobiological pathways responsible for euphoria and strong reinforcing effects, thereby may substantially reducing abuse liability. At the same time, intrinsic μ-receptor antagonism may modify the adverse-effect profile of these agents, potentially contributing to reduced risks of respiratory depression and constipation, and producing a characteristic ceiling effect on respiratory depression (14).

For agents that combine *κ*-receptor agonism with partial μ-receptor agonism, the ceiling effect of μ-mediated analgesia arises fundamentally from the limited intrinsic efficacy of partial agonists. Even when receptor occupancy is maximal, the capacity of these agents to activate μ receptors remains inherently constrained and cannot achieve the maximal maximal effect produced by high-efficacy μ-receptor agonists (e.g., the selective agonist fentanyl, or the non-selective classical agonist morphine). This ceiling phenomenon does not reflect a transition to pure antagonism at higher doses; rather, it represents the natural pharmacodynamic plateau associated with full receptor occupancy by a partial agonist (4). However, when co-administered with high-efficacy agonists, high doses of partial agonists may competitively displace these full agonists due to their receptor affinity, thereby producing functional antagonism. Together, these pharmacological properties are thought to underlie the distinctive clinical profile of opioid agonist–antagonists, which combine effective analgesia with an improved safety margin (5).

Because opioid agents differ in receptor subtype affinity and pharmacological activity, opioid receptor agonist–antagonists exhibit diverse analgesic effects and adverse-event profiles. Currently marketed agents in this class primarily include *κ*-receptor agonists with μ-receptor partial agonist or antagonist activity, as well as μ-receptor agonists with *κ*-receptor partial agonist or antagonist properties (15, 16).

## 3 Combination of opioid agonists with *κ*-agonist/μ-partial agonist or antagonist agents

### 3.1 Pentazocine

Pentazocine was the first opioid receptor agonist–antagonist introduced into clinical practice and exhibits a characteristic dose-dependent profile involving multiple opioid receptor subtypes. The drug primarily acts as a *κ*-opioid receptor agonist while displaying dual activity as a partial agonist and weak antagonist at the μ-opioid receptor; at higher doses, activation of *δ* receptors has also been reported (17). The psychotomimetic effects (e.g., hallucinations, dysphoria) associated with pentazocine are primarily attributed to *κ*-receptor activation (Tables 1, 2).

**Table 1:** Pharmacodynamic comparison of *κ*-agonist/μ-partial agonist or antagonist used alone.

Pharmacodynamic parameter	Pentazocine	Butorphanol	Nalbuphine
Receptor profile	*κ* agonist + μ partial agonist/antagonist + weak *δ* agonist; *σ* receptor activity	Strong *κ* agonist + μ partial agonist/antagonist	Strong *κ* agonist + μ antagonist/weak agonist
Analgesic potency (vs. morphine)	Low. Oral/injectable ≈ 1/3–1/4 morphine; low ceiling	High. Injectable ≈ 5–8× morphine (≈5–7 mg ≈10 mg morphine)	Moderate. Injectable ≈ morphine (10 mg ≈10 mg morphine)
Analgesic ceiling	Present. IM > 30–40 mg plateau	Present. IV > 2–3 mg plateau	Present. IV > 10–20 mg plateau
Respiratory depression	Dose-related with ceiling effect; weaker than others	Clear ceiling effect; lower than morphine at equianalgesic doses	Clear ceiling effect; lower than morphine at equianalgesic doses
Cardiovascular effects	May increase BP and HR; increases cardiac load	Minimal effect	Minimal; may slightly reduce BP
Psychotomimetic effects	Common (agitation, nightmares, hallucinations)	Present (sedation, dizziness); lower than pentazocine	Lowest incidence; more sedation, less agitation
μ antagonism strength	Moderate; may precipitate withdrawal	Weak to moderate	Strong; reverses opioid side effects without full analgesia reversal
GI motility	Less inhibition than high-efficacy μ-receptor agonists	Less severe constipation	Weaker than high-efficacy μ-receptor agonists
Biliary pressure	May increase	Significantly increases; contraindicated in acute biliary disease	Significantly increases; contraindicated in acute biliary disease

**Table 2:** Combination of κ-agonist/μ-partial agonist or antagonist with high-efficacy μ-receptor agonists.

Parameter	Pentazocine + μ agonist	Butorphanol + μ agonist	Nalbuphine + μ agonist
Analgesic interaction	Unpredictable; often antagonistic; not routinely recommended	Complex; synergy in opioid-naïve; antagonism in high-dose μ exposure	Complex; strong μ antagonism may partially reverse analgesia
Respiratory depression	May reduce but introduces other risks	Possible stabilizing effect; evidence inconsistent	Commonly used to reverse respiratory depression
Pruritus	May reduce	Highly effective	Highly effective
Sedation/agitation	Higher agitation risk	Increased sedation	Converts euphoria to calmer sedation
Clinical recommendation	Avoid routine combination	Use with close monitoring	Primarily rescue therapy for adverse effects

Early studies using systemic administration in multiple murine pain models demonstrated that the analgesic effect of low-dose pentazocine (3–30 mg/kg) was completely abolished by the selective μ-receptor antagonist C-CAM but was unaffected by the *κ*-receptor antagonist nor-BNI, indicating that low-dose analgesia is primarily mediated by partial μ-receptor activation (18). More recent intrathecal studies at the spinal level in rats showed that the analgesic effect of low-dose pentazocine can be blocked by both nor-BNI and the μ-receptor antagonist CTAP, suggesting that both *κ* and μ receptors contribute to spinal analgesia at low doses, with *κ*-receptor activation not representing the dominant mechanism (19).

When the systemic dose is increased to 56–100 mg/kg, the analgesic efficacy of pentazocine declines, producing a bell-shaped dose–response curve (20). Further experiments demonstrated that pretreatment with nor-BNI enhances the analgesic effect at high doses and converts the bell-shaped curve into a sigmoidal pattern, indicating that *κ*-receptor activation at higher doses may counteract μ-receptor–mediated analgesic pathways (18, 20). This bidirectional inter-receptor antagonism and negative modulation is thought to result in a plateau of analgesic efficacy, thereby potentially generating a ceiling effect. Consistent with this interpretation, Ide et al. (21) reported that in μ-receptor knockout mice, pentazocine produced a dose-dependent reduction in analgesic efficacy for somatic pain, yielding a biphasic dose–response pattern (21). In contrast, in models of visceral chemical pain, analgesic effects were preserved in μ-receptor knockout animals and were completely abolished by nor-BNI, indicating that visceral analgesia is predominantly mediated by *κ*-receptor activation and differs mechanistically from somatic pain modulation (21).

Given its dual pharmacological profile of *κ*-receptor agonism and μ-receptor partial agonism/antagonism, pentazocine exhibits clear dose-dependent interactions when combined with potent μ-opioid receptor agonists, a phenomenon observed in both experimental and clinical studies. Preclinical data demonstrate potential risks associated with high-dose combinations: high-dose pentazocine (10–100 mg/kg, subcutaneous) antagonizes the analgesic effect of high-dose morphine (10 mg/kg) (20). Clinical evidence further supports a dose-dependent interaction pattern. In elderly patients undergoing thoracoscopic surgery, administration of pentazocine 1.5 mg/kg combined with sufentanil 1.2 μg/kg during anesthetic induction achieved analgesia comparable to sufentanil 2.5 μg/kg alone while significantly reducing adverse effects such as nausea, vomiting, and somnolence (22, 23). In contrast, in patients undergoing esophageal cancer surgery, postoperative patient-controlled intravenous analgesia using pentazocine (total dose 120 mg) combined with sufentanil (100 μg) provided adequate analgesia but was associated with a high incidence of nausea, vomiting, and dizziness (24).

Although these studies differ in species, surgical context, and dosing regimens, collectively they support a consistent conclusion: at low to moderate doses, pentazocine combined with sufentanil may achieve synergistic analgesia and reduced adverse effects likely through complementary receptor mechanisms, a strategy that may be particularly advantageous in elderly patients; however, at higher doses, the μ-receptor antagonistic activity of pentazocine may predominate, leading to increased adverse effects and diminished clinical benefit (25). Accordingly, precise dose selection is critical in clinical practice, and optimization and standardization of dosing strategies across clinical contexts require further well-designed studies.

### 3.2 Butorphanol

Butorphanol is a potent *κ*-opioid receptor agonist with a characteristic activity profile across opioid receptor subtypes (*κ*:μ:*δ* ≈ 25:4:1) and exhibits distinctive μ-receptor partial agonist–antagonist properties (26). Upon occupying μ receptors, butorphanol produces only limited partial agonist activity; however, in the presence of potent μ-opioid agonists, its relatively high receptor affinity enables competitive inhibition of full agonist binding, thereby producing functional antagonism (27). This pharmacological profile confers several clinically relevant advantages. Analgesia is thought to be achieved through synergistic spinal mechanisms mediated by *κ*-receptor activation together with partial μ-receptor agonism, while intrinsic μ-receptor antagonism may attenuate excessive depression of the respiratory center, thereby reducing the risk of respiratory depression (28). Ye et al. demonstrated that even after intravenous administration of 30 μg/kg butorphanol, respiratory depression was limited to a reversible reduction in respiratory parameters within approximately 7 min of injection and exhibited a clear ceiling effect (29).

With respect to dependence liability, animal models of dependence and withdrawal reported by Jaw et al. and Commiskey et al. indicate that *κ*-receptor activation by butorphanol suppresses mesolimbic dopaminergic reward pathways, and its limited μ-receptor intrinsic efficacy results in substantially lower physical dependence compared with conventional μ-opioid agonists (26). In the prevention of opioid-induced hyperalgesia, studies in laparoscopic surgery demonstrated that patients receiving high-dose remifentanil exhibited significantly increased postoperative visual analog scale (VAS) scores after extubation, whereas co-administration of butorphanol 0.02 mg/kg effectively attenuated hyperalgesia (30). Kong et al. further confirmed in a randomized double-blind trial that low-dose butorphanol (0.2 μg/kg) significantly reduced both postoperative VAS scores and fentanyl consumption associated with high-dose remifentanil exposure (31).

Predominant *κ*-receptor activity also has been associated with reduced μ-receptor–mediated inhibition of gastrointestinal motility and smooth muscle spasm, while μ-receptor antagonistic properties may decrease mast cell degranulation, potentially lowering the incidence of pruritus and urinary retention (32). Compared with high-efficacy μ-opioid agonists, butorphanol demonstrates superior efficacy in visceral pain control and is associated with lower rates of nausea, vomiting, dizziness, and respiratory depression (33).

When combined with high-efficacy μ-receptor agonists, butorphanol has been reported to exhibit significant synergistic analgesic effects together with improved safety, likely through complementary receptor subtype activity. In combination with potent μ-receptor agonists, *κ*-receptor activation enhances spinal analgesia, while partial agonist–antagonist modulation of μ-receptor function may preserve baseline analgesia and simultaneously help limit excessive μ-receptor activation–related adverse effects, possibly through competitive mechanisms (34). In clinical practice, He et al. demonstrated that a regimen of butorphanol 5 mg combined with fentanyl 1.0 mg diluted to 100 mL and administered as a background infusion of 2 mL/h with patient-controlled bolus dosing provided stable postoperative analgesia and significantly reduced nausea, vomiting, and dizziness compared with single-agent therapy (35). Yao et al. further optimized dosing in postoperative analgesia after cesarean section, showing that intravenous infusion of butorphanol 240 μg/h (equivalent to 12 mg/50 mL) combined with epidural morphine 2 mg maintained effective analgesia while reducing pruritus from 25% to 0% and nausea and vomiting from 40% to 10% (36). Qian et al. explored a novel regimen using a loading dose of oxycodone 0.25 mg/kg combined with butorphanol 0.1 mg/kg followed by background infusion and patient-controlled analgesia, demonstrating particularly effective control of uterine contraction pain after repeat cesarean delivery and earlier onset of lactation (37).

Collectively, these findings indicate that rational combination of butorphanol with opioid receptor agonists achieves synergistic modulation of *κ* and μ receptors, overcoming the limited analgesic spectrum of single-agent therapy while substantially reducing typical opioid-related adverse effects such as respiratory depression and pruritus. This receptor-balanced strategy provides an important pharmacological basis for individualized and precision analgesia in clinical practice.

### 3.3 Nalbuphine

The pharmacological activity of nalbuphine is based on its dual action at μ and *κ* opioid receptors, characterized primarily by μ-receptor antagonism and *κ*-receptor agonism (38). Early systematic reviews reported that intravenous nalbuphine administered at doses of 0.3–0.5 mg/kg provides effective analgesia and exhibits a pronounced ceiling effect on respiratory depression, such that further dose escalation beyond a certain plasma concentration does not produce additional respiratory suppression. Comparative clinical data between nalbuphine and morphine further demonstrated significantly lower incidences of pruritus, nausea, and vomiting with nalbuphine (39–41).

Beyond common adverse effects, nalbuphine also demonstrates unique clinical value in the management of specific opioid-related complications. Ibrahim et al. reported a case of urinary retention induced by hydromorphone in which a single intravenous dose of nalbuphine 10 mg resulted in successful voiding of 850 mL within 6 h without compromising analgesia. This observation suggests that nalbuphine may reverse opioid-induced urinary retention through μ-receptor antagonism while maintaining analgesia via *κ*-receptor activation (42).

In combination therapy, multiple studies have highlighted the complex pharmacodynamic interactions between nalbuphine and other opioid agents. Yeh et al. demonstrated that the addition of nalbuphine (1 mg/mL) to morphine-based patient-controlled intravenous analgesia (PCIA) maintained comparable analgesia while significantly reducing μ-receptor–related adverse effects such as nausea and vomiting (43). A dose-finding study by Yang et al. further showed that nalbuphine 0.4 mg/kg combined with sufentanil 2.0 μg/kg significantly reduced postoperative effective demand frequency and decreased the incidence of nausea and vomiting from 30% to 3%, indicating an optimal dose balance. However, when the nalbuphine dose was increased to 0.8 mg/kg, analgesic efficacy remained similar but the incidence of somnolence increased to 33%, suggesting a dose-related risk profile (44).

These findings are consistent with studies by Xiang et al., which collectively suggest that nalbuphine may provide compensatory analgesia through spinal *κ*-receptor activation, partially offsetting its antagonistic effects at μ receptors (45). Nevertheless, Wong et al. reported that co-administration of nalbuphine 5 mg with morphine did not produce the anticipated synergistic enhancement of analgesia, indicating that variations in dose combinations may alter the balance of μ and *κ* receptor activity and thereby influence clinical outcomes (46).

Overall, the interaction between nalbuphine and μ-receptor agonists exhibits a clear dose-dependent pattern. Appropriate dose combinations appear to preserve analgesic efficacy while being associated with significantly reducing adverse effects, whereas excessive dosing may increase sedation due to predominant *κ*-receptor activation (47). These findings underscore the importance of precisely modulating receptor balance in clinical practice and support individualized dosing strategies to optimize both analgesic efficacy and safety.

## 4 Combination of opioid agonists with buprenorphine and dezocine

### 4.1 Buprenorphine

Buprenorphine is a pharmacologically distinctive opioid that functions as a high-affinity partial agonist at the μ-opioid receptor while producing clinical analgesic efficacy comparable to that of high-efficacy μ-receptor agonists (48). This apparent paradox may be explained by its multimodal receptor activity. An expert consensus reported by Webster et al. (49) characterized buprenorphine as a *κ*-receptor antagonist, *δ*-receptor antagonist, and full agonist at the ORL-1 (nociceptin) receptor, with coordinated multi-receptor activity contributing to sustained analgesic efficacy without a ceiling effect for analgesia (49) (Tables 3, 4).

**Table 3:** Pharmacodynamic comparison of buprenorphine and dezocine used alone.

Pharmacodynamic parameter	Buprenorphine	Dezocine
Classification & receptor action	μ receptor partial agonist; δ/κ receptor antagonist; ORL-1 (nociceptin) receptor full agonist; high affinity and slow dissociation	κ receptor partial agonist; μ receptor partial agonist; weak norepinephrine/5-HT reuptake inhibition
Analgesic potency	Very high. Potency vs. morphine: sublingual ≈ 100–150:1; injectable ≈ 70–100:1	Moderate. IV ≈ 1/2–1/3 morphine (5–10 mg ≈10 mg morphine)
Analgesic characteristics	Strong analgesia; slow onset (IV ∼ 15 min); long duration (6–8 h)	Rapid onset (IV 2–5 min); moderate duration (3–5 h)
Analgesic ceiling effect	No clear ceiling for analgesia; ceiling effect present for respiratory depression	Marked; no further increase beyond single dose > 0.2 mg/kg
Respiratory depression	Present with ceiling effect; difficult to reverse with naloxone; nonlinear dose relationship	Ceiling effect; may be lower than morphine at equianalgesic doses
Cardiovascular effects	Mild; possible slight bradycardia	May increase blood pressure and heart rate
Sedation/psychological effects	Marked sedation; weak euphoria; agitation rare	Sedation and somnolence common; low agitation incidence
μ receptor binding characteristics	Very high affinity; slow dissociation; displaces other opioids	Moderate affinity; partial agonist
Gastrointestinal effects	Constipation less than high-efficacy μ agonists	Weaker than high-efficacy μ-receptor agonists
Dependence & withdrawal	Low dependence potential; mild delayed withdrawal	Lower dependence than high-efficacy μ-receptor agonists
Special pharmacokinetics	Low sublingual bioavailability (∼30%); strong first-pass metabolism	Low oral bioavailability; mainly injectable use
Main clinical uses	Moderate-to-severe pain; opioid substitution therapy; chronic pain	Postoperative analgesia; moderate-to-severe acute pain

**Table 4:** Pharmacodynamic comparison of buprenorphine and dezocine combined with high-efficacy μ-receptor agonists.

Parameter	Buprenorphine + μ agonist	Dezocine + μ agonist
Analgesic interaction	Highly complex and may be antagonistic due to high μ receptor affinity	Possible synergy but requires caution; μ partial agonism may antagonize pure agonist
Effect on respiratory depression	Complex and risky; may reduce depression but difficult naloxone reversal	May reduce respiratory depression due to ceiling effect
Modulation of side effects	May reduce pruritus and nausea but increase sedation	May reduce μ agonist–induced pruritus via κ activation
Pharmacokinetic interaction	Key pharmacodynamic interaction: prolonged μ receptor occupancy (hours–days)	No significant PK interaction; mainly pharmacodynamic
Clinical recommendation	Avoid routine combination; if required, higher μ agonist dose may be needed	Cautious use in multimodal analgesia with close monitoring
Special considerations	Naloxone resistance; transdermal users may require 2–5× opioid intraoperatively	Limited high-quality evidence; individualized dosing required

Notably, a systematic review and randomized controlled meta-analysis by Stanley demonstrated endpoint-specific partial agonism: buprenorphine does not exhibit a dose ceiling for analgesia and may achieve complete pain relief with dose escalation, whereas a clear ceiling effect is present for respiratory depression, thereby enhancing clinical safety (50). This differential profile may reflect pathway-selective signaling mechanisms. Buprenorphine preferentially activates *β*-arrestin–mediated signaling in respiratory-related neurons, which is associated with lower functional efficacy, while analgesic effects may be mediated predominantly through G protein–dependent pathways. Regional differences in receptor density, such as higher μ-receptor expression in pain-processing pathways compared with brainstem respiratory centers, may further contribute to this phenomenon (51).

Regarding the potential antagonistic effects associated with partial agonists, experimental evidence from Kögel et al. provides important mechanistic insight. At conventional analgesic doses (e.g., buprenorphine 0.0316 mg/kg i.v. combined with morphine 1 mg/kg i.v.), buprenorphine produced additive or synergistic analgesic effects with high-efficacy μ-receptor agonists (e.g., the selective agonist fentanyl, or the non-selective classical agonist morphine). Antagonistic effects were observed only at supratherapeutic doses corresponding to the descending limb of a bell-shaped dose–response curve (e.g., buprenorphine 4.64–6.81 mg/kg i.p.), at which competitive receptor binding reduced morphine-induced analgesia (21.5 mg/kg i.p.) to levels comparable to buprenorphine monotherapy (12).

Taken together, buprenorphine's unique multi-receptor pharmacological profile and pathway-selective signaling confer synergistic analgesic potential with high-efficacy μ-receptor agonists at clinically relevant doses while maintaining a ceiling effect on respiratory depression. Antagonistic interactions attributable to partial agonism appear to occur only under experimental conditions involving doses substantially exceeding those used in clinical practice.

### 4.2 Dezocine

Dezocine, a synthetic opioid widely used in the Chinese analgesic market, has undergone a gradual clarification of its pharmacological identity, evolving from early ambiguity to a more defined receptor profile centered on its intrinsic activity at opioid receptors. Initial studies classified dezocine as a *κ*-receptor agonist based on its analgesic efficacy, whereas subsequent *in vitro* investigations (e.g., Gharagozlou et al.; Liu et al.) suggested that it might function as a *κ*-receptor antagonist (52, 53). This apparent discrepancy was critically addressed by Wang et al., who demonstrated through functional assays that dezocine produces only weak activation of both *κ*- and μ-receptor–mediated G protein signaling, with maximal efficacy substantially lower than that of high-efficacy μ-receptor agonists (e.g., the selective agonist fentanyl, or the non-selective classical agonist morphine). On this basis, dezocine was characterized as a partial agonist (54).

Importantly, in the presence of high-efficacy μ-receptor agonists, dezocine exhibits competitive inhibitory effects consistent with functional antagonism. This pharmacological pattern, weak agonism when administered alone and antagonistic behavior in the presence of high-efficacy μ-receptor agonists, accords with the classical definition of partial agonism. Accordingly, dezocine can be defined as a *κ*-opioid receptor partial agonist and a μ-opioid receptor partial agonist–antagonist. At low to moderate analgesic doses, dezocine produces analgesia through moderate activation of *κ* and μ receptors, but its intrinsic efficacy exhibits a ceiling effect at approximately 10 mg/kg. When potent μ-receptor full agonists are present, such as high-dose morphine in clinical therapy, dezocine may competitively occupy μ receptors and potentially attenuate excessive receptor activation, thereby contributing to reducing adverse effects associated with high-efficacy μ-receptor agonists, including respiratory depression, nausea, and vomiting. This pharmacological profile provides a mechanistic basis for its potential to mitigate opioid-related toxicity in combination therapy (55, 56).

Clinical evidence supports these pharmacodynamic observations. Early studies demonstrated that postoperative analgesic regimens combining sufentanil 100 μg with dezocine 20 mg, as well as dezocine 0.25 mg/kg combined with sufentanil 1.5 μg/kg, provided satisfactory analgesia in patients undergoing radical esophagectomy and abdominal oncologic surgery while significantly reducing the incidence of nausea, vomiting, somnolence, and dizziness, highlighting the toxicity-sparing effect of dezocine in combination therapy (24, 57). A recent prospective randomized controlled study further evaluated the effect of dezocine on the median effective dose (ED50) of sufentanil required to induce respiratory depression under spinal anesthesia. The combined regimen increased the ED50 of sufentanil to 0.342 μg/kg (95% CI: 0.269–0.623) compared with 0.291 μg/kg (95% CI: 0.257–0.346) with sufentanil alone, indicating that dezocine increases the dose threshold required to induce respiratory depression without increasing severe adverse events. These findings suggest that combination therapy broadens the safety window of sufentanil and enhances perioperative analgesic safety (55).

Collectively, the combination of dezocine with sufentanil engages multiple analgesic pathways while reducing the required dose of sufentanil through receptor-level modulation. This dose-sparing effect directly decreases the incidence of sufentanil-related adverse effects, including respiratory depression and dependence liability, while also reducing dezocine-related adverse effects such as somnolence by limiting the need for high-dose monotherapy.

## 5 Conclusion

This review provides an integrated analysis of the pharmacological principles and clinical application strategies of opioid receptor agonist–antagonists, thereby clarifying three key conceptual questions that motivated this work.

First, regarding whether μ-receptor antagonism retains clinical value when these agents are used alone, the answer is unequivocally affirmative. Intrinsic μ-receptor antagonism is considered a central pharmacological advantage. By competitively occupying and functionally blocking receptor sites responsible for intense euphoria and reinforcement, these agents inherently reduce abuse liability. Simultaneously, this mechanism is believed to underlie the characteristic ceiling effect observed in respiratory depression and related dose-limiting adverse effects, thereby enhancing clinical safety. In addition to μ-receptor antagonism, *κ*-receptor activation, particularly at supraspinal sites, can produce dysphoric effects that functionally oppose μ-receptor-mediated euphoria, further contributing to the reduced abuse liability of these agents.

Second, with respect to the origin of the ceiling effect of μ-receptor partial agonists and its relationship to antagonistic activity, the present synthesis indicates that the analgesic ceiling primarily is thought to reflect an intrinsic property of partial agonism, namely, limited intrinsic efficacy. The affinity profiles of common opioid drugs for the major opioid receptor subtypes are summarized in Table 5. Once receptor occupancy reaches saturation, pharmacodynamic responses plateau without transformation into pure antagonism. However, when combined with selective high-efficacy μ-receptor agonists (e.g., sufentanil, fentanyl) or non-selective classical agonists (e.g., morphine), the high receptor affinity of partial agonists enables competitive displacement of high-efficacy μ-receptor agonists, resulting in functional antagonism at the receptor level. This interaction exhibits a clear dose-dependent pattern. At low-to-moderate combination doses (e.g., butorphanol 0.02–0.1 mg/kg combined with sufentanil or fentanyl), complementary *κ*-receptor activation and μ-receptor modulation may produce synergistic analgesia, particularly for visceral pain, while being associated with significantly reducing μ-related adverse effects including respiratory depression, nausea, vomiting, and pruritus. In contrast, excessive dosing (as observed with pentazocine in certain experimental settings) may allow μ-receptor antagonism to predominate, thereby attenuating high-efficacy μ-receptor agonist–mediated analgesia or introducing new adverse effects such as excessive sedation. These findings underscore the critical importance of dose optimization and highlight the need for rigorously designed studies to define optimal dose ratios across pain models and patient populations in order to precisely balance receptor-level synergy and antagonism.

**Table 5:** Affinity profiles of common opioid drugs for major opioid receptor subtypes.

Drug category	Representative drug	μ Receptor (MOR)	κ Receptor (KOR)	δ Receptor (DOR)	Key characteristics & notes
Strong Agonist	Fentanyl	Very High Affinity (Ki ∼ 0.1–1 nM)	Low Affinity	Low Affinity	Selective μ receptor agonist, potency approximately 80–100 times that of morphine.
Strong Agonist	Sufentanil	Very High Affinity (Ki ∼ 0.01 nM)	Low Affinity	Low Affinity	Selective μ receptor agonist, highest affinity in the fentanyl family, most potent (approx. 1,000 times morphine).
Classic Agonist	Morphine	High Affinity (Ki ∼ 1–10 nM)	Moderate Affinity (Ki ∼10–50 nM)	Low Affinity (Ki > 100 nM)	Prototypical drug, relatively selective for μ receptor, but not absolute.
Classic Agonist	Pentazocine	Moderate Affinity (Ki ∼ 5–20 nM)	Strong Agonist, High Affinity (Ki ∼ 0.5–5 nM)	Agonist, Moderate Affinity (Ki ∼ 5–20 nM)	Has clear agonistic activity at δ receptor (DOR); however, its psychotomimetic effects (hallucinations, dysphoria) are primarily attributed to κ-receptor activation.
Classic Agonist	Hydromorphone	High Affinity (Ki ∼ 0.5–5 nM, slightly > morphine)	Similar to or slightly higher than morphine (Ki ∼ 10–50 nM)	Low Affinity	Potency approximately 6–8 times that of morphine, receptor profile similar to morphine.
Classic Agonist	Oxycodone	Low to Moderate Affinity, requires metabolic activation (Ki > 100 nM)	Relatively high κ affinity (Ki ∼ 50–500 nM)	Low Affinity	Its analgesic effect is thought to involve both μ and κ receptors; κ activity may contribute to its unique side effect profile.
Partial Agonist	Buprenorphine	Very High Affinity, Partial Agonist (Ki ∼ 0.1–1 nM)	Antagonist/Weak Agonist	Antagonist	High-affinity, partial agonist at μ receptor; ceiling effect (respiratory depression only; analgesia shows no clear ceiling); used for analgesia and medication-assisted treatment for opioid use disorder
Partial Agonist	Dezocine	High Affinity, but Partial/Weak Antagonist (Ki ∼ 1–10 nM)	High Affinity (Ki ∼ 0.5–3 nM)	Low Affinity (Ki > 100 nM)	κ activity provides primary analgesia; μ partial agonism offers some synergistic analgesia and euphoria while limiting respiratory depression and addiction potential, but also poses risks in opioid-tolerant patients.
Agonist-Antagonist	Butorphanol	Partial Agonist/Weak Antagonist (Ki ∼ 1–10 nM)	Strong Agonist (Ki ∼ 0.5–5 nM)	Weak Agonist/Antagonist	Primarily acts as a κ receptor agonist and μ receptor partial agonist/antagonist, providing analgesia with lower risk of μ-mediated euphoria and respiratory depression.
Agonist-Antagonist	Nalbuphine	Antagonist (Ki ∼ 1–10 nM)	Strong Agonist (Ki ∼ 0.5–5 nM)	Unknown/Weak Agonist	Pure κ receptor agonist and μ receptor antagonist, does not produce μ-mediated euphoria.
Pure Antagonist	Naloxone	High Affinity Antagonist (Ki ∼ 1 nM)	High Affinity Antagonist	High Affinity Antagonist	Non-selective antagonist, highest affinity for μ receptor, used to reverse opioid overdose.
Pure Antagonist	Naltrexone	High Affinity Antagonist	High Affinity Antagonist	High Affinity Antagonist	Long-acting non-selective antagonist, orally effective.

Notably, while the dual-receptor framework provides a useful conceptual model for understanding the clinical profile of opioid agonist-antagonists, it is important to acknowledge that the precise contribution of each receptor component and whether comparable analgesic efficacy and safety margins could be achieved with μ-selective partial agonists alone, remains incompletely defined. The existing literature provides suggestive evidence for *κ*-mediated contributions to certain effects, such as the attenuation of μ-mediated euphoria and pruritus, but direct comparative studies between dual-acting agents and μ-selective partial agonists are limited. We therefore emphasize that the mechanistic interpretations offered in this review should be viewed as hypotheses that require further validation through specifically designed preclinical and clinical investigations. Future research should prioritize head-to-head comparisons to delineate the distinct roles of μ vs. *κ* receptor engagement in determining the overall therapeutic profile of these agents.

Collectively, opioid receptor agonist–antagonists establish a pharmacological intermediate between pure agonists and pure antagonists, providing an essential framework for balanced analgesia in clinical practice. The emergence of functionally selective ligands represents the next evolutionary direction, aiming to achieve signaling-level dissociation within the μ receptor itself. This review proposes a three-dimensional conceptual framework integrating receptor subtype selectivity, dose, and pharmacodynamic response, and provides a theoretical basis for future development of a dynamic dose–effect balance model of opioid receptor subtype–selective activation. Whether through optimization of existing agonist–antagonist combination strategies or through refinement of biased agonist pharmacology, the shared objective remains the achievement of effective analgesia with minimized adverse effects.
